# Prolonged Indirect Hyperbilirubinemia as a Manifestation of Postnatally Acquired Cytomegalovirus Infection

**DOI:** 10.7759/cureus.98217

**Published:** 2025-12-01

**Authors:** Rita Alvelos, Tomás Ferrão, Katia Mauricio, Vitor Coutinho, Maria Miguel Abreu Almiro

**Affiliations:** 1 Pediatrics, Local Health Unit of the Aveiro Region (ULSRA), Aveiro, PRT; 2 Primary Health Care, USF Flor de Sal, Aveiro Health Centre, Local Health Unit of the Aveiro Region (ULSRA), Aveiro, PRT

**Keywords:** cytomegalovirus (cmv), neonatal jaundice, postnatal cmv infection, prolonged hyperbilirubinemia, unconjugated hyperbilirubinemia

## Abstract

Prolonged neonatal jaundice is defined as jaundice lasting more than 14 days. Cytomegalovirus (CMV) is a ubiquitous pathogen that may present in young infants as a postnatally acquired infection, frequently transmitted through breast milk.

We report the case of a two-month-old infant who presented with prolonged indirect hyperbilirubinemia (no other relevant hepatic alterations) associated with neutropenia. Abdominal ultrasound and Gilbert syndrome testing were unremarkable. Polymerase chain reaction (PCR) testing for CMV in urine was positive at three months of age, while the Guthrie card blood sample testing, taken at three days of age, was negative.

Postnatally acquired CMV infection is frequently asymptomatic in immunocompetent infants, although it may manifest with prolonged hyperbilirubinemia and transient hepatic enzyme elevation. In literature, it is more commonly associated with conjugated hyperbilirubinemia. This case highlights the importance of considering CMV infection as a potential cause of isolated prolonged indirect jaundice, as well as emphasizing its typically benign course and prognosis.

## Introduction

Prolonged neonatal jaundice is defined as jaundice lasting more than 14 days in full-term neonates, or more than 21 days in preterm newborns [1]. The most common etiologies differ depending on whether cholestasis is present. In cases of prolonged unconjugated jaundice, the most common causes include breast milk jaundice, hemolysis (due to Rh or AB0 incompatibility, extravasated blood, or glucose-6-phosphate dehydrogenase deficiency), congenital hypothyroidism, and urinary tract infection [2,3].

Cytomegalovirus (CMV) is a DNA virus and a member of the Herpesviridae family. It is a ubiquitous pathogen, with most infections in immunocompetent hosts being asymptomatic or presenting as a mononucleosis-like syndrome, which may include CMV hepatitis [4,5]. Congenital CMV infection results from transplacental transmission and can present with significant involvement of various systems. In addition to congenital transmission, young infants might acquire CMV peri- or postnatally, through contact with maternal cervicovaginal secretions during delivery, breast milk, and blood transfusions or close contact with family members, most frequently toddlers in the household [6,7]. Among these possible transmission routes, breast milk is considered the most common in infants born to CMV-IgG seropositive mothers [8]. While CMV is a recognized cause of neonatal hepatitis and cholestatic jaundice, it may rarely be associated with elevated levels of unconjugated bilirubin [5]. Distinguishing these two forms is essential, as their natural history and implications for investigation differ. Although PCR testing of Guthrie card dried blood spots is a valuable retrospective tool for identifying congenital infection, a negative result cannot definitively exclude congenital CMV, due to its low sensitivity.

We report the case of an infant with prolonged indirect jaundice due to postnatally acquired CMV infection.

## Case presentation

A two-month-old infant was observed in the emergency department for vomiting, which started on the day prior to observation and occurred after every meal. He also presented with more frequent bowel movements and decreased intake. No fever or other symptoms were mentioned. He was exclusively breastfed with excellent weight gain and did not usually have reflux. There was no significant family history nor current acute illness among his household contacts.

Concerning personal history, he was a firstborn child, from a well-monitored pregnancy, with all recommended ultrasounds showing no pathological findings and serological tests yielding unremarkable results, aside from evidence of prior maternal CMV exposure documented both in the first and third trimesters (negative immunoglobulin M and positive immunoglobulin G). The mother presented with fever during labor, which warranted antibiotic therapy. The infant was born by caesarean section at 39 weeks and 6 days of gestation and did not require resuscitation. Anthropometric measurements at birth were as follows: weight, 4045 g (percentile 85); height, 51 cm (percentile 49); and head circumference, 35 cm (percentile 51). Neonatal screenings, including the newborn endocrine and metabolic screening test and audiologic screening, were normal. He passed urine and meconium within the first 24 hours of life, both with normal characteristics. During his stay in the maternity unit, he was diagnosed with neonatal jaundice, with a total serum bilirubin level of 19.89 mg/dL (reference values: 0.3-1.2). He underwent phototherapy between the third and fourth days of life, after which the total serum bilirubin value decreased to 12.51 mg/dL. His blood group was A, Rh-positive, with a negative direct antiglobulin (Coombs) test.

Physical examination during the ED episode was unremarkable except for icteric coloration of the sclerae, facial, and trunk skin. Analytical study (Table 1) revealed neutropenia (0.7 x 10^9^/L; reference values: 1.0-8.5 x 10^9^) with otherwise normal leucogram, hemoglobin, and platelets. It also documented hyperbilirubinemia (10.7 mg/dL; reference values: 0.3-1.2) at the expense of indirect bilirubin (10.1 mg/dL; reference values: 0.1-1.0). Gamma-glutamyl transferase (GGT) and lactate dehydrogenase (LDH) were slightly elevated (91 U/L; reference values: GGT, <73 and 284 U/L and LDH, 120-246 U/L). Transaminases and alkaline phosphatase were within normal values. Renal function, serum electrolytes, and C-reactive protein (CRP) were also unremarkable, and fecal virus antigen testing was negative for rotavirus and adenovirus. The hypothesis of acute gastroenteritis associated with breast milk jaundice was considered. Oral tolerance was tested in the emergency room, and he was discharged on a fractionated diet and a probiotic.

**Table 1:** Analytical results at the first emergency room admission

Laboratory parameter	Value	Reference range	Interpretation
Hemoglobin (g/dL)	11	9.5-13.5	Normal
Reticulocytes (x10^9^/L)	67	50-100	Normal
Neutrophils (x10^9^/L)	0.7	0.1-8.5	↓↓
Lymphocytes (x10^9^/L)	4.2	4.0-10.5	Normal
Platelets (x10^9^/L)	286	200-550	Normal
Alanine aminotransferase (U/L)	44	10.0-49.0	Normal
Aspartate aminotransferase (U/L)	61	8.0-69.0	Normal
Total bilirubin (mg/dL)	10.7	0.3-1.2	↑↑
Indirect bilirubin (mg/dL)	10.1	0.1-1.0	↑↑
Gamma-glutamyl transferase (U/L)	91	<73.0	↑
Lactate dehydrogenase (U/L)	284	120-246	↑
Alkaline phosphatase (U/L)	351	107-431	Normal
Reactive C protein (mg/dL)	<0.05	0.00-0.50	Normal

At three months of age, he was seen in consultation by his general practitioner (GP) for sustained jaundice, which had persisted since his discharge from maternity. He maintained exclusive breastfeeding and normal weight gain, as well as unchanged stool and urine appearance and pattern. There were no new dietary or pharmacological exposures. On physical examination, there were no significant alterations except for a slight icteric coloration of the sclerae, face, and trunk.

**Figure 1:**
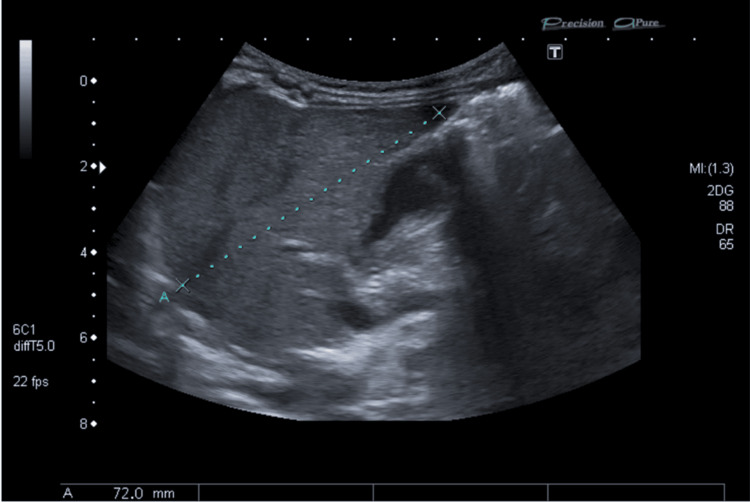
Abdominal ultrasound Abdominal ultrasound shows the liver of normal size, with regular contours, homogeneous echostructure, and normal reflectivity. It measures approximately 72 mm in the longitudinal axis in the right lobe. There is no dilation of the intra- and extrahepatic bile ducts. No suggestive signs of biliary atresia (such as the absence of a normal gallbladder and presence of triangular cord sign) or anatomical alterations were present. The absence of biliary dilation ruled out obstructive hyperbilirubinemia.

He was referred to the emergency department, where an analytical study was repeated, showing maintained, although improved, neutropenia (0.8 x 10^9^/L) with otherwise normal full blood count. Blood smear revealed the presence of some reactive lymphocytes. Unconjugated hyperbilirubinemia persisted with total bilirubin of 3.9 mg/dL and indirect bilirubin of 3.1 mg/dL. Liver enzymes were within normal values. Renal function, serum electrolytes, and CRP were also unremarkable. Abdominal ultrasound did not show any relevant alterations (Figure 1). A genetic study for Gilbert’s syndrome was performed and showed heterozygosity for TA6/TA7, which is not typically described as being associated with Gilbert’s syndrome. A CMV PCR was carried out on a urine sample, evidencing 8756 copies/mL (any virus detection is considered abnormal). CMV PCR on the Guthrie card blood sample, which had been sampled on the third day of life, was negative.

There was progressive clinical improvement, with no specific therapy or other pharmacological exposures except for recommended supplementation with vitamin D, maintaining exclusive breastfeeding. Jaundice resolved at around four months of age. At follow-up consultation after four weeks, blood workup was normal, except for neutropenia (0.9 x 10^9^/L), attributed to viral infection.

Currently, the patient is a toddler, maintaining follow-up with his GP. He remains asymptomatic with no history of recurrent infections and is fulfilling all developmental milestones.

## Discussion

Neonatal jaundice is a common condition that frequently affects newborn babies, presenting as a yellowish coloring of the skin and sclerae due to the accumulation of bilirubin [9]. Although it is typically benign and transient, prolonged jaundice, defined as jaundice lasting more than 14 days in full-term babies or more than 21 days in pre-term babies, might have a more severe etiology and requires further investigation to exclude pathology. The 2023 NICE Guidelines recommendations for prolonged jaundice include assessing for pale stools and/or dark urine, measuring conjugated bilirubin, carrying out a full blood count, blood group determination (mother and baby), and a direct antiglobulin test. It also suggests considering a urine culture if there is clinical suspicion of a urinary tract infection and to ensure that routine metabolic screening (including screening for congenital hypothyroidism) has been performed [1]. However, some have questioned the need for such an extensive workup, as in most infants it uncovers no significant pathology. Additionally, it results in significant cost, heightened parental anxiety, and may negatively impact breastfeeding. A stepwise approach might be feasible, with full liver function and investigating the underlying cause only after confirming direct hyperbilirubinemia [10-12], as it usually implicates a more serious condition [2,13-15]. Yet, any infant who remains jaundiced beyond two to three weeks of age should have the serum bilirubin level fractionated [16].

In this case, the presentation with prolonged indirect hyperbilirubinemia, without additional significant hepatic alterations, would suggest etiologies such as breast milk jaundice, hemolysis, congenital hypothyroidism, and urinary tract infection [2,3]. These etiologies were excluded through the exclusion of anemia and AB0 incompatibility, a normal blood smear, negative RCP, and normal neonatal metabolic screening.

CMV infection in young infants can be acquired congenitally, perinatally, or postnatally [5] and is a known potential cause of neonatal hyperbilirubinemia. It may evolve with viral hepatitis, possibly due to the hepatic immaturity characteristic of this age group [9]. The transmission rate of CMV has been reported as 11.1% via perinatal routes [17]. Other than ingestion or aspiration of cervicovaginal secretions at the time of delivery, one of the most common and most efficient routes of perinatal transmission is through ingestion of fresh breast milk, with breastfed infants showing a higher rate of infection when compared to formula-fed infants [4,8]. CMV is reactivated during lactation in up to 70%-95% of CMV-seropositive mothers and is frequently shed in their breast milk, especially in mothers with high levels of CMV IgG antibody. As many as 53% of children breastfed with milk containing infectious virus or CMV DNA can become infected [4,17]. This type of milk-borne CMV infection apparently protects children from CMV disease, and CMV seropositivity may provide protection against CMV inclusion disease in the next generation [10]. Postnatally acquired CMV infection does not cause obvious adverse events in infants born over 32 gestational weeks. Therefore, detection of CMV DNA in breastmilk should not be routinely performed, and breastfeeding should be recommended [8].

In order to distinguish congenital from postnatal infection, PCR should be performed on a sample collected within three weeks of birth, ideally as soon as possible after birth [18]. In the present case, negative PCR on the Guthrie card reduced the likelihood of congenital infection. However, a negative result cannot definitively exclude congenital CMV, as its sensitivity has been described at around 84% [18].

The spectrum of disease caused by CMV infection in pediatric patients ranges from asymptomatic to potentially life-threatening diseases [4]. Preventing CMV infection is challenging due to the virus’s ubiquity. Currently, prevention relies on maintaining adequate hygiene practices [18]. Perinatally acquired infections in healthy infants usually manifest between 4 and 16 weeks of age [4] and, unlike congenital CMV infection that can lead to extensive liver involvement, are generally not symptomatic (due to maternal antibody transfer across the placenta to the fetus) [17]. Postnatally acquired CMV infection may not lead to clinically meaningful liver involvement. In a cohort of 384 infants, ALT abnormalities occurred at similar rates in CMV-infected and CMV-uninfected infants (around 15%), and no cases of significant hepatitis or cholestasis were observed [8]. However, about one-third of infants may present transiently with lymphadenopathy, hepatosplenomegaly, hepatitis, pneumonitis, and abnormal blood counts or liver function tests [4,19]. Postnatally acquired CMV infection has been described as a cause of prolonged neonatal jaundice associated with cholestasis and elevated ALT levels, in asymptomatic, well-thriving infants, without urine or stool alterations [10]. Nevertheless, like in the present case, another study has reported high indirect bilirubin levels in around 47% of patients presenting with CMV hepatitis, with prolonged jaundice and abdominal distension as the most common complaints [5]. It is hypothesized that hepatic inflammation and subsequent transient enzyme dysfunction, even if mild, may play a role in delaying bilirubin metabolism and excretion [19].

Recognizing CMV infection as a potential, but uncommon, cause of prolonged indirect jaundice is clinically important, as early diagnosis may guide appropriate follow-up and management. Studies suggest there are no obvious adverse events after acquired CMV infection in immunocompetent infants born over 32 gestational weeks, including no effects on long-term growth, neurodevelopment, or liver enzyme levels [8,17]. In fact, mildly elevated transaminases can be a subclinical finding and can resolve spontaneously without the need for treatment in two to three months [5,10].

In the present case, the detection of CMV in an immunocompetent infant with prolonged indirect hyperbilirubinemia is in line with the literature that describes a benign presentation of postnatal CMV infection. The decision to treat CMV hepatitis with antivirals, such as ganciclovir, is controversial in immunocompetent children and generally is reserved for severe conditions with involvement of other systems [5]. The absence of other significant liver alterations and the benign course, as observed in this case, reinforce the idea that postnatal CMV infection has a good prognosis.

## Conclusions

In conclusion, this case illustrates the importance of considering CMV infection as a potential cause of prolonged indirect hyperbilirubinemia, even in the absence of other evident hepatic alterations.

The spontaneous recovery witnessed, as in most cases of CMV hepatitis in immunocompetent infants, highlights the frequent favorable course of disease. Additionally, it emphasizes the need for a thorough differential diagnosis in prolonged jaundice cases, while offering a reassuring perspective on the prognosis of presumably postnatally acquired CMV infection in otherwise healthy infants.
